# Preferential Entry of Botulinum Neurotoxin A Hc Domain through Intestinal Crypt Cells and Targeting to Cholinergic Neurons of the Mouse Intestine

**DOI:** 10.1371/journal.ppat.1002583

**Published:** 2012-03-15

**Authors:** Aurélie Couesnon, Jordi Molgó, Chloé Connan, Michel R. Popoff

**Affiliations:** 1 Institut Pasteur, Unité des Bactéries anaérobies et Toxines, Paris, France; 2 CNRS, Institut de Neurobiologie Alfred Fessard – FRC2118, Laboratoire de Neurobiologie– et Développement UPR3294, Gif sur Yvette, France; University of Illinois, United States of America

## Abstract

Botulism, characterized by flaccid paralysis, commonly results from botulinum neurotoxin (BoNT) absorption across the epithelial barrier from the digestive tract and then dissemination through the blood circulation to target autonomic and motor nerve terminals. The trafficking pathway of BoNT/A passage through the intestinal barrier is not yet fully understood. We report that intralumenal administration of purified BoNT/A into mouse ileum segment impaired spontaneous muscle contractions and abolished the smooth muscle contractions evoked by electric field stimulation. Entry of BoNT/A into the mouse upper small intestine was monitored with fluorescent HcA (half C-terminal domain of heavy chain) which interacts with cell surface receptor(s). We show that HcA preferentially recognizes a subset of neuroendocrine intestinal crypt cells, which probably represent the entry site of the toxin through the intestinal barrier, then targets specific neurons in the submucosa and later (90–120 min) in the musculosa. HcA mainly binds to certain cholinergic neurons of both submucosal and myenteric plexuses, but also recognizes, although to a lower extent, other neuronal cells including glutamatergic and serotoninergic neurons in the submucosa. Intestinal cholinergic neuron targeting by HcA could account for the inhibition of intestinal peristaltism and secretion observed in botulism, but the consequences of the targeting to non-cholinergic neurons remains to be determined.

## Author Summary

Botulism is a severe and often fatal disease in man and animals characterized by flaccid paralysis. *Clostridium botulinum* produces a potent neurotoxin (botulinum neurotoxin) responsible for all the symptoms of botulism. Botulism is most often acquired by ingesting preformed botulinum neurotoxin in contaminated food or after intestinal colonization by *C. botulinum* under certain circumstances, such as in infant botulism, and toxin production in the intestine. The first step of the disease consists in the passage of the botulinum neurotoxin through the intestinal barrier, which is still poorly understood. We investigated the trafficking of the botulinum neurotoxin in a mouse intestinal loop model, using fluorescent HcA (half C-terminal domain of the heavy chain). We observed that HcA preferentially recognizes neuroendocrine intestinal crypt cells, which likely represent the entry site of the toxin through the intestinal barrier, then targets specific neurons, mainly cholinergic neurons, in the submucosa, and later (90–120 min) in the musculosa leading to local paralytic effects such as inhibition of intestinal peristaltism. These results represent an important advance in the understanding of the initial steps of botulism intoxication and can be the basis for the development of new specific countermeasures against botulism.

## Introduction

Botulinum neurotoxins (BoNTs) are responsible for a severe nervous disease in man and animals known as botulism, characterized by skeletal muscle flaccid paralysis and respiratory arrest, resulting from inhibition of acetylcholine (ACh) release in peripheral cholinergic nerve terminals. BoNTs are produced by *Clostridium botulinum* as single chain proteins (ap. 150 kDa), which are divided into 7 toxinotypes (A to G) according to their immunogenic properties. The toxins are exported outside the bacteria and are proteolytically cleaved into a heavy chain (H; ap. 100 kDa) and a light chain (L; ap. 50 kDa), which remain linked by a disulfide bridge. The di-chain molecule constitutes the active neurotoxin. The half C-terminal domain of the H-chain (Hc) is involved in binding to specific receptors on target neuronal cells and in driving the toxin entry pathway into cells, whereas the N-terminal part permits the translocation of the L chain into the cytosol. The L chain catalyzes a zinc-dependent proteolysis of one or two of the three proteins of the SNARE complex, which play an essential role in evoking neurotransmitter exocytosis. The BoNT/A L-chain cleaves the synaptosomal associated protein SNAP25 at the neuromuscular junction [1]–[4]. The highly specific binding of BoNTs to target nerve endings involves protein and ganglioside receptors that localize at the neuronal plasma membrane [5]. Gangliosides of GD_1b_ and GT_1b_ series are involved in binding and functional entry into cells of BoNT/A and BoNT/B [6]–[9]. The protein receptors on neuronal cells have been identified as synaptotagmin I and II for both BoNT/B and BoNT/G, and synaptic vesicle protein SV2 (isoforms A, B and C) for BoNT/A [8], [10]–[14], BoNT/E [15], and BoNT/F [16], [17]. SV2C is the preferred BoNT/A neuronal receptor [14], whereas BoNT/E recognizes glycosylated SV2A and SV2B [15]. BoNT/D also uses SV2 proteins as receptor in association with gangliosides for its entry into neuronal cells, but binds to SV2 via a distinct mechanism than BoNT/A and BoNT/E [18]. In addition, SV2A and SV2B have also been evidenced to mediate the entry of tetanus toxin (TeNT) into the central target neurons including hippocampal and spinal cord neurons [19].

Botulism usually results from the ingestion of preformed neurotoxin in contaminated food, or ingestion of spores or bacteria, which under certain circumstances, may colonize the gut and produce the neurotoxin *in situ* [20]. In either case, BoNT escapes the gastro-intestinal tract to reach the target cholinergic nerve endings, possibly through the blood and lymph circulation [21]. Indeed, previous observations have shown that after oral administration of BoNT in experimental animals, the toxin enters the blood and lymph circulation. The upper small intestine was found to be the primary site of absorption [22]–[25], but BoNT can also be absorbed from the stomach [21]. Penetration of BoNT through an epithelial cell barrier and its subsequent migration to cholinergic nerve endings are the essential first steps of botulinum intoxication. In *in vitro* models, BoNTs have been found to bind to polarized epithelial cells and to undergo receptor-mediated endocytosis and transcytosis from apical to basolateral sides [24], [26]–[30]. However, little is known about the precise pathway of BoNT migration from the intestinal lumen to the target nerve endings.

The digestive tract contains its own independent nervous system, the enteric nervous system (ENS), which is as complex as the central nervous system, and it is also referred as the “brain of the gut”. ENS controls and coordinates motility, exocrine and endocrine secretions, and blood microcirculation of the gastrointestinal tract. Nerve cell bodies of ENS are clustered into small ganglia which are organized in two major plexuses: the myenteric plexus between the longitudinal and circular muscle layers, and the submucosal plexus associated with the mucosal epithelium between the circular muscles and the muscularis mucosa. Ganglia also contain glial cells and their extensions. ENS neurons can be classified as afferent sensory neurons, interneurons, and motor neurons, which are connected to the central autonomic nervous system through both sensory and motor pathways. More than 20 types of neurotransmitters have been identified in ENS, and most enteric neurons may produce and release several of them. However, neurotransmitter functions have not been fully identified. Secretory and motor neurons are cholinergic, these latter also contain substance P. Myenteric neurons are connected to the cholinergic parasympathetic neurons through nicotinic, and in some areas, muscarinic receptors [31], [32]. Vasoactive intestinal protein (VIP) and serotonin are also major neurotransmitters in the regulation of normal gut function and interconnection with the central nervous system [33].

In this study, we used fluorescent Hc fragment from BoNT/A to monitor the trafficking of the toxin into the mouse intestinal mucosa. It has been previously shown that the Hc domain from TeNT, which shares similar structural organization and catalytic activity with BoNTs, is a useful tool to investigate the intracellular trafficking of the neurotoxin [34]–[36]. Although, it cannot be ruled out that a cross interplay between the BoNT domains may modify the toxin routing driven by Hc [37], recombinant HcA has been reported to retain the same structure than that of the receptor binding domain of the BoNT/A holotoxin and to enter hippocampal neurons similarly to the whole neurotoxin [16], [38]. In addition, HcA has been found to bind and transcytose through intestinal cells as well as the holotoxin [26], [39], validating its use to investigate the intestinal trafficking of the toxin.

## Results

### Effect of BoNT/A on muscle tension from isolated ileum segments

In striated muscle, BoNT/A is known to inhibit ACh release from motor nerve terminals by cleaving the synaptosomal associated protein SNAP-25, leading to the inability of synaptic vesicles containing ACh to undergo transmitter release [for a review, see [3]]. In the gastrointestinal smooth muscle, BoNT/A also impairs cholinergic transmission by inhibiting ACh release from postganglionic cholinergic nerve endings *in vitro* and *in vivo* [40], [41]. The first aim of this study was to determine whether BoNT/A affected smooth muscle contractility when applied intra-luminally on isolated mouse ileum segments.

**Figure 1:**
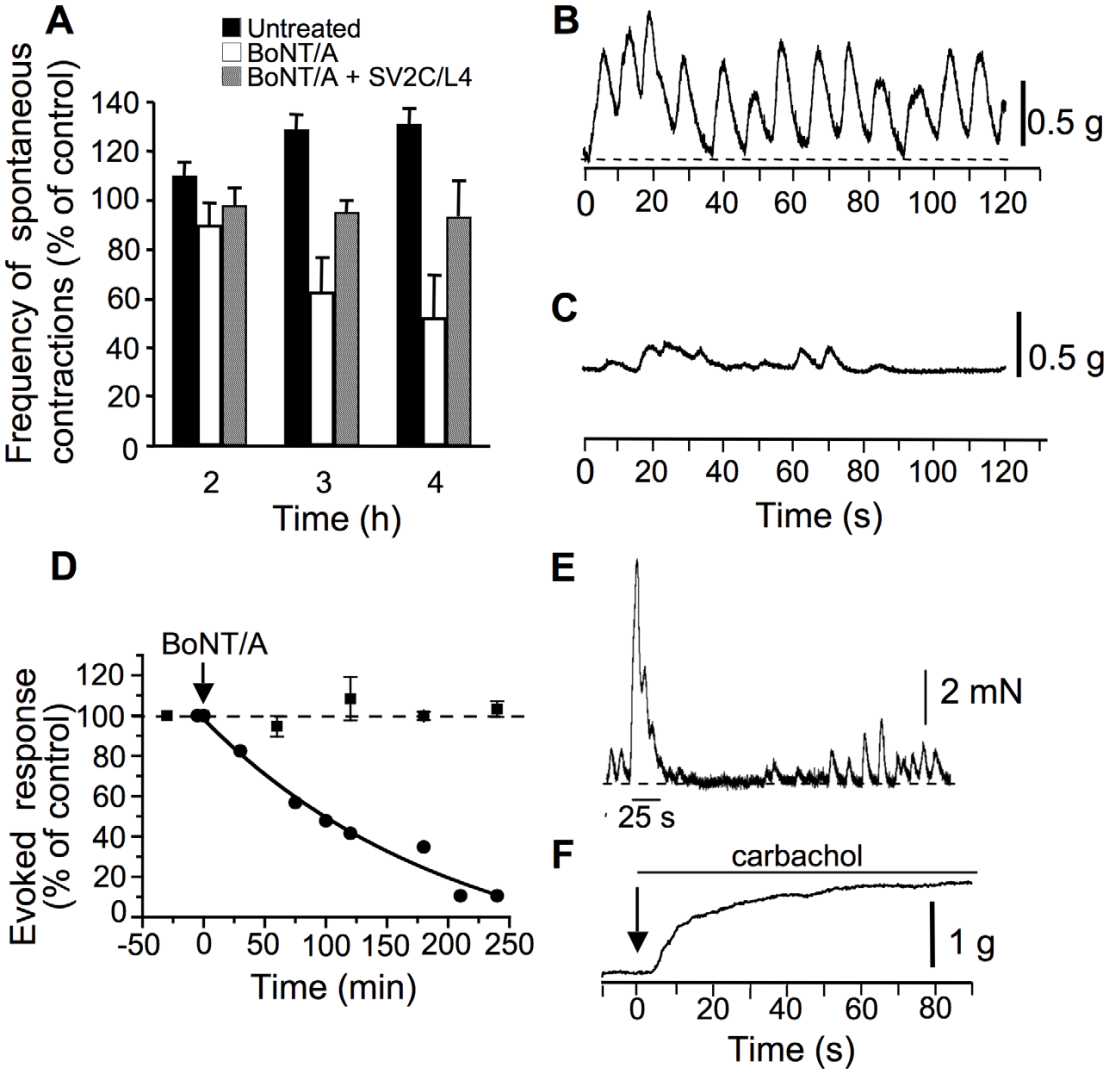
Effect of BoNT/A on longitudinal isometric muscle contraction from mouse ileal segments. (**A**) Spontaneous ileal contraction frequency as a function of time under control conditions (black colums), and after BoNT/A (10^5^ LD_50_/ml) injection into the lumen of ligated mouse ileal segments (white columns). BoNT/A (10^5^ LD_50_/ml) was preincubated with GST-SV2C/L4 (20 µg/ml for 30 min at room temperature prior to being inoculated into ligated intestinal loop. Examples of spontaneous contractions before (**B**), and after 4 h of BoNT/A treatment (**C**). Note that BoNT/A significantly decreased, but not completely the frequency and amplitude of the spontaneous contractile activity. Data are from 3 independent experiments. (**D**). Time-dependent reduction of electrically-evoked longitudinal muscle contractions after BoNT/A injection into intestinal lumen. (**E**) Representative continuous control recording showing spontaneous contractions before and after single electrical field stimulation (30 Hz for 25 s, indicated by the trace below the evoked recording). Note the changes in spontaneous contractions following the evoked one. (**F**) In ileal segment treated for 4 h with BoNT/A, carbachol (20 µM) applied to the external medium (arrow) induced a sustained muscle contraction, indicating that ACh receptors were still functional. The line segment above the tension record indicates the time of carbachol application.

In preparations that were equilibrated in the standard oxygenated solution for about 30 min, spontaneous contractile responses were usually observed at a frequency rate of about 6–10 min^−1^ (n = 4). These spontaneous contractions had a peak force that was variable from preparation to preparation, but comprised between 0.5 and 1.2 g (n = 4). BoNT/A reduced their frequency after 2, 3 and 4 h of the intralumenal injection (Figure 1A). It is worth noting that for control preparations maintained for 4 h in the same conditions as the ones treated with BoNT/A, not only there was no reduction of the spontaneous contractions, but a small increase in their frequency (10% after 2 and 3 h) (Figure 1A). The contraction pattern changed also after exposure to BoNT/A, from very regular oscillations during the first hour to very irregular contractions after more than 2 h (Figure 1B and 1C).

Electric field stimulation evoked contractile responses that attained a peak force comprised between 2 and 7 mN (n = 3) under control conditions (Figure 1E), with little rundown of the responses when stimulations were applied every 50–60 min (Figure 1D). As shown in Figure 1D, BoNT/A reduced the electrically-evoked contractile response in a time-dependent manner. The time to decrease to 50% the evoked tension-time integral response was about 110 min, and the toxin reduced to about 90% electrically-evoked contractions within 240 min. Although it is difficult to exclude the possibility of direct muscle stimulation by the applied field-stimuli, several lines of evidence indicate that this would represent no more than 10–15% of the evoked tension-time integral response in our experimental conditions. Most of this evidence comes from: (i) Data obtained with BoNT/A showing that blockade of the evoked contraction attains a maximum around 86–90% of control values, and was never complete. The remaining tension can be suspected to be due to direct muscle stimulation unaffected by BoNT/A. (ii) If direct stimulation of the muscle would occur, one would expect that tension levels would be sustained and maintained during the field stimulation, which is not the case (Figure 1E). (iii) Spontaneous contractions occurring during the falling phase of the evoked-contractile response were not enhanced in amplitude, which is consistent with the low influence of direct muscle stimulation under the experimental conditions used. Interestingly, after BoNT/A has blocked the evoked response by field stimulation, the addition of carbachol (20 µM) or ACh (data not shown) to the standard solution evoked a contractile response (Figure 1F). These results suggest that under the conditions used BoNT/A is able to exert an action on cholinergic terminals that leads to a blockade of the contractile responses evoked by electric field stimulation, while spontaneous myogenic contractions were reduced in frequency, but not completely abolished. The fact that carbachol could induce contractile activity after BoNT/A-induced blockade of contraction evoked by electric field stimulation, strongly suggests that the sensitivity of ACh receptors (muscarinic and nicotinic) is not affected by the toxin (Figure 1F).

In a previous report, we have found that BoNT/A transcytosis through intestinal cell monolayers grown on filters is mediated by SV2C or, at least, an immunologically related protein [30]. To address whether SV2C might be a functional receptor in the *ex vivo* intestinal tract model, BoNT/A was preincubated with the intravesicular domain segment L4 of SV2C prior to injection into ligated intestinal loop. As shown in Figure 1A, preincubation with SV2C/L4 significantly prevented the BoNT/A inhibitory effects on spontaneous intestinal smooth muscle contractions, suggesting a partially SV2C-dependent BoNT/A uptake through the intestinal mucosa. However, these results do not rule out that SV2C/L4 also passed, independently or associated with BoNT/A, through the intestinal barrier and impaired BoNT/A uptake by nerve terminals.

### Distribution of HcA binding sites in the mouse small intestine

**Figure 2:**
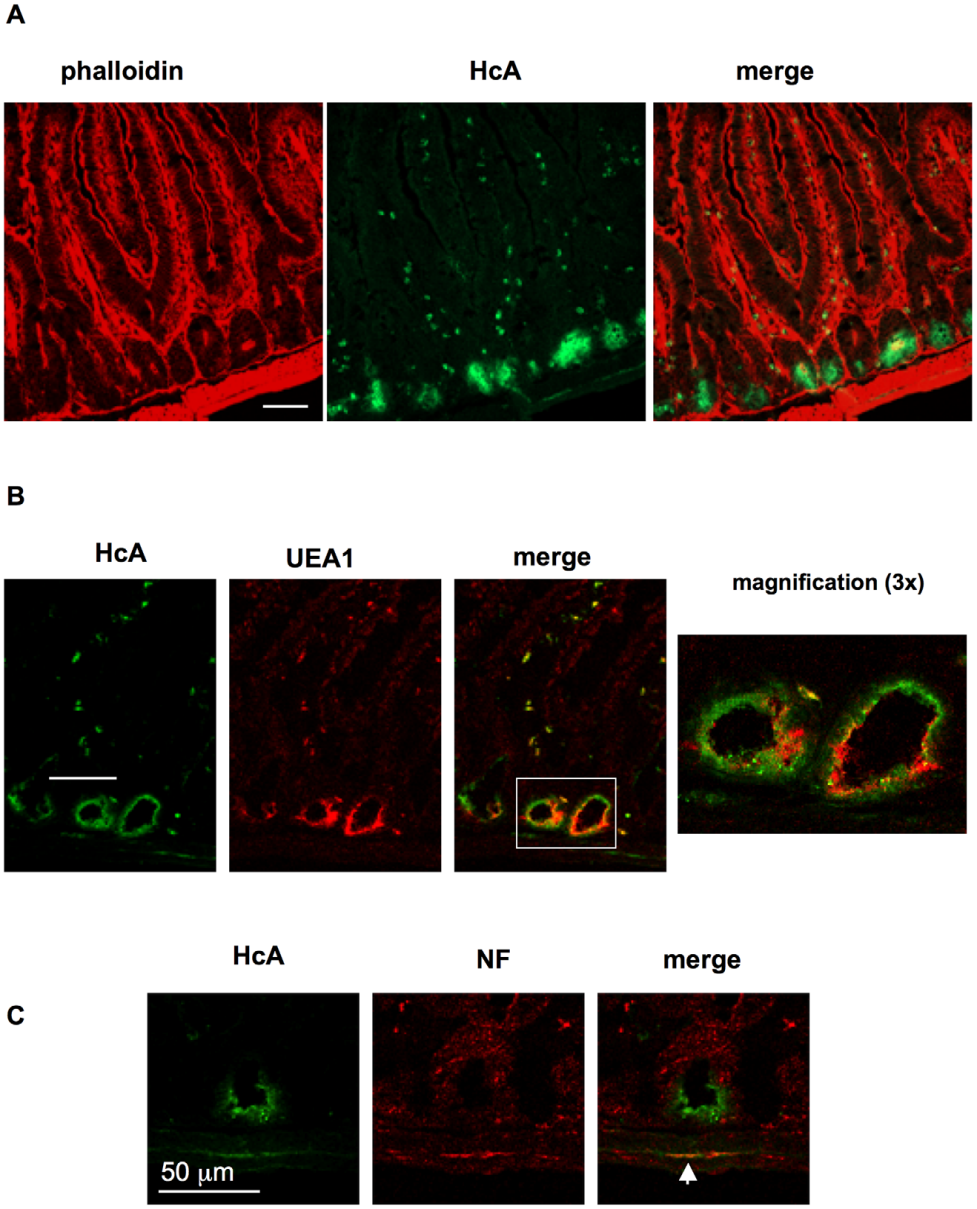
Binding of HcA to frozen sections of the mouse upper small intestine. (**A**) Sections were overlaid with Alexa488-HcA and were counterstained with TRITC-Phalloidin. HcA staining (green) was observed in intestinal crypts, small cells in intestinal villi beneath the enterocytes, as well as in filaments from the submucosa. (**B**) Counterstaining with TRITC-UEA1 (red) labeling of Paneth cells in intestinal crypts and small cells in intestinal villi. (**C**) Counterstaining with anti-neurofilament (NF) antibodies and co-labelling (arrowhead) of neurofilaments with HcA in the submucosa. (scale bars = 50 µm).

We first investigated the potential binding sites for HcA in mouse intestine mucosa and submucosa, as well as in the musculosa. For that, cryosections of mouse small intestine, fixed on glass slides, were incubated with fluorescent HcA and analyzed by confocal microscopy. Since BoNT has been reported to be preferentially absorbed from the upper small intestine [23], [24], sections from ileum were analyzed. Only a faint staining was observed in brush border of enterocytes along intestinal villi (Figure 2A). However, a strong HcA binding was observed on intestinal crypt localized at the bottom of villi. Paneth cells, which are characterized by their numerous secretory-granule content, are spatially restricted to intestinal crypts and were used as a marker of these regions. Staining of Paneth cells with the TRITC-labeled lectin *Urex europaeus* agglutinin type 1 (UEA1) [42] did not significantly colocalize with HcA (Figure 2B). This may indicate that some cells from intestinal crypts, distinct from Paneth cells, exhibit preferential binding sites for HcA. Moreover, small cells scattered along the villi were stained with HcA (Figure 2A) and some of them co-stained with UEA1 (Figure 2B). These UEA1 positive cells likely correspond to goblet cells [43].

It is worth noting that HcA stained neuronal cell bodies and neuronal structures in the submucosa and musculosa. However, only a low proportion of neuronal structures were recognized and labeled by HcA, as revealed by immunolabeling neurofilaments and co-staining with the fluorescent toxin fragment (Figure 2C). Hence, the binding domain of BoNT/A potentially targets epithelial cells in intestinal crypts, and neuronal structures of intestinal plexuses. Note that anti-neurofilament antibodies are not specific of the nerve endings, where BoNT/A is assumed to bind, but recognize neuronal structures all along the neuronal cells. This probably accounts for the irregular co-staining between HcA and anti-neurofilament antibodies. In addition, the irregular pattern of HcA staining might also be related to the variability in orientation and size of the cryosections. Moreover, cryosections might artificially expose certain antigens which are buried in intact tissues. Thus, immunostaining pattern in cryosections has to be considered with caution and confirmed in *ex vivo* experiments with intact tissues as shown in the following figures.

### Entry of HcA into mouse intestinal mucosa

**Figure 3:**
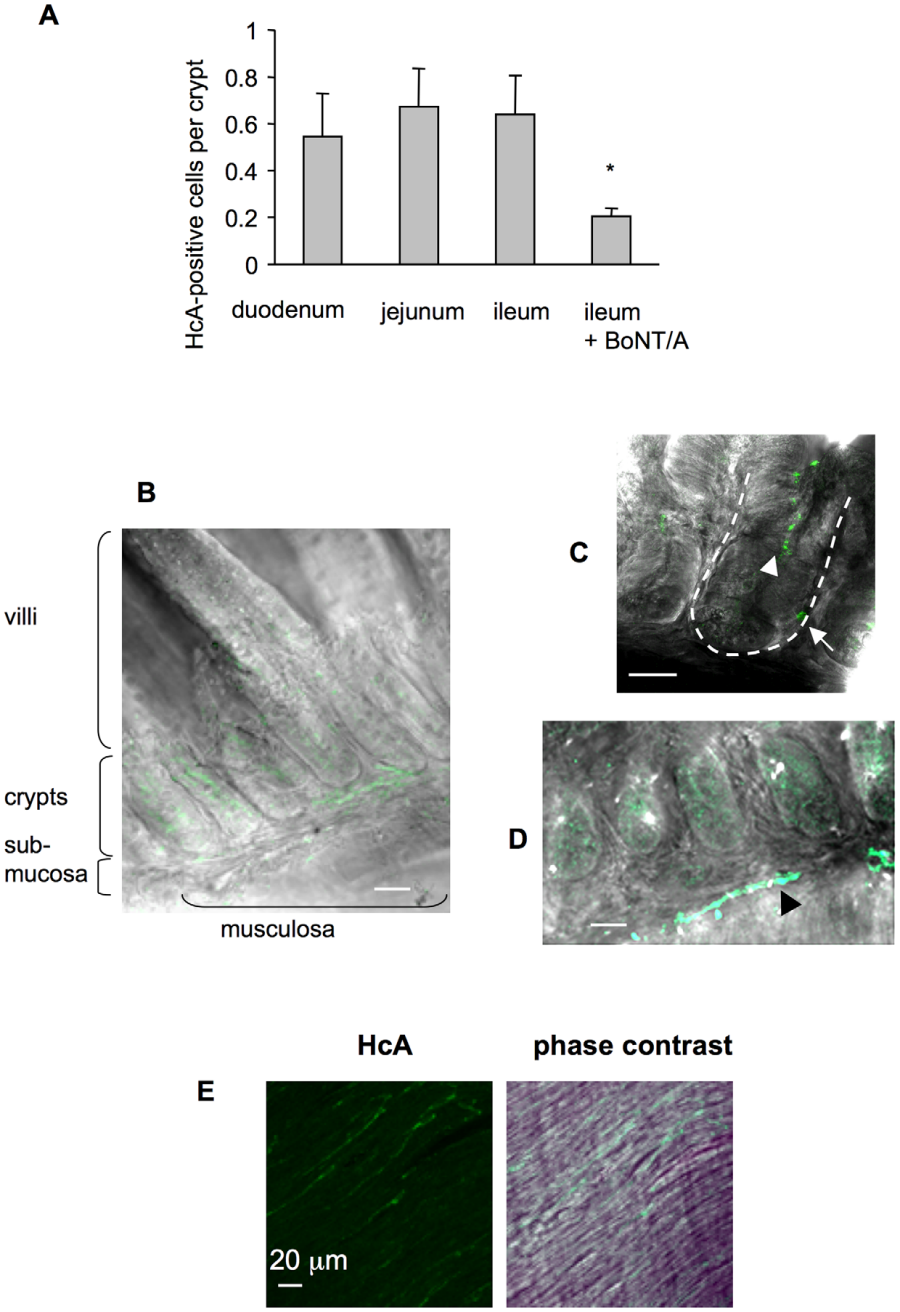
Visualization of HcA in the mouse intestine following inoculation into the intestinal lumen. (**A**) HcA-Cy3 (0.5 µg) was injected into ligated ileum, duodenum, or jejunum loop. After 30 min of incubation, the samples were fixed and the mucosal fluorescence was measured as the number of HcA-positive cells per crypt. The number of labeled cells was similar between the three intestinal segments, but significantly lower (*) in the competition assay with BoNT/A (2.5 µg) in an ileal loop (Student's t test). (**B**) Detection of HcA (green) in the intestinal crypts and in some submucosa areas. No or only weak labeling is observed in the intestinal villi. (**C**) Visualization of HcA in intestinal crypt lumen (arrow-head) and inside some intestinal crypt cells (arrow), and (**D**) on filaments in the submucosa (black arrow head) after 30 min incubation. (**E**) HcA labeling of filaments (corresponding to neurofilaments, see Figure 7) in the musculosa after 90 min incubation. (scale bars = 20 µm).

To analyze HcA entry into the intestinal mucosa and submucosa, *ex vivo* experiments were performed, as previously described with the whole toxin. For this, excised small intestine loops were washed, ligated at both extremities, and incubated in oxygenated Krebs-Ringer solution at 37°C. Fluorescent HcA was inoculated into the intestinal lumen, and at various time intervals, intestinal loops were washed, fixed and processed for dissection, and immunostaining. A competition assay between HcA-Cy3 and native BoNT/A injected into an ileum loop and monitored by fluorescence analysis of the intestinal mucosa, supported that fluorescent HcA follows the same entry pathway than native BoNT/A (Figure 3A). Fluorescent HcA entered similarly ileum, duodenum or jejunum segments, as tested by mucosal fluorescence analysis (Figure 3A). After 30–60 min incubation, labeled HcA was detected inside the lumen of intestinal crypts, and in some crypt cells, but not or with a low intensity in enterocytes or other cells in the villi (Figure 3 B and C). HcA also labeled long cell extensions in the submucosa, which correspond to nerve fibers or neuronal extensions (Figure 3D; arrow head) since they were co-stained with antibodies against neurofilaments (not shown). Longer incubation periods (90–120 min) permitted to visualize HcA staining of long filaments in the musculosa, (Figure 3E), which were identified as nerve fibers from the myenteric plexus (see below), but with a weaker intensity. This suggests a progressive entry of HcA from the intestinal lumen through the mucosa, preferentially through intestinal crypts, to certain neuronal cell and extensions in the submucosa, and then in the musculosa.

### HcA preferentially targets intestinal neuroendocrine crypt cells

**Figure 4:**
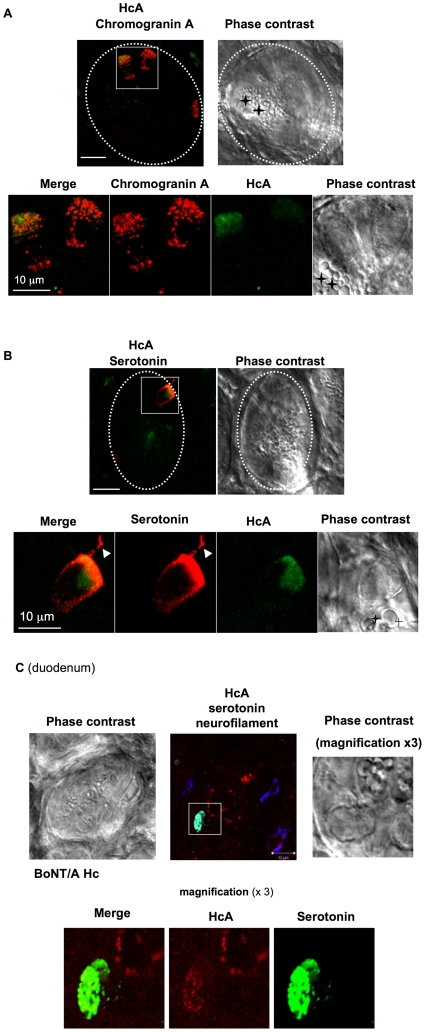
Visualization of HcA in neuroendocrine cells from mouse intestinal crypts. Fluorescent HcA (0.5 µg) was injected into the lumen of a mouse ileum (**A, B**) or duodenum (**C**) loop, and after 30 min incubation the intestinal loop was washed and prepared for immunostaining with chromogranin A or serotonin antibodies. (**A**) Two cells stained with chromogranine A antibodies in an intestinal crypt (dotted circle) were co-labeled with HcA (green). Magnification of one cell (square) shows a uniform punctuate distribution of chromogranin A staining, whereas HcA was preferentially localized at the basal pole. Phase contrast shows that the chromogranin A-immunoreactive cell contained no large granules, in contrast to Paneth cell (black cross). (**B**) Co-labeling of a cell from an intestinal crypt (dotted circle) with HcA (green) and serotonin antibodies (red). Magnification of the cell (square) shows a basal distribution of both HcA and serotonin. Note that a cell extension was also labeled with serotonin antibodies but not by HcA (arrowhead), and that the serotonin-immunoreactive cell contained no large granules as in Paneth cells (black cross) (scale bars = 10 µm). (**C**) Co-labeling of HcA (red) with serotonin (green) in a duodenum crypt cell. Neurofilament staining (blue) was observed at the crypt periphery.

To identify the intestinal crypt cells targeted by HcA, fluorescent HcA was injected into the lumen of an intestinal loop. After an incubation of 15–30 min, the intestinal mucosa was prepared for microscopy observation. Only few numbers (1 or 2) of small cells from each intestinal crypt were stained with HcA (Figure 4). Cells stained with HcA were distinct from Paneth cells, which were easily detectable by their numerous granules (Figure 4), and by their staining with UEA1 (not shown). Chromogranin-A antibodies, a common marker of neuroendocrine cells in the gastrointestinal tract [44], colocalized with HcA (Figure 4A). All cells stained with HcA were also stained with chromogranin-A antibodies, indicating that HcA specifically entered neuroendocrine cells from intestinal crypts. However, not all chromogranin-A positive cells were stained with HcA, but only about 80%. Serotonin-producing cells, which are abundant in the ENS, were investigated for their colocalization with HcA. In the intestinal crypts, all the cells stained with HcA were also immunolabeled with serotonin antibodies (Figure 4B and C). Interestingly, HcA accumulated in the basal pole of neuroendocrine cells, which is wider than the apical pole exposed to the intestinal crypt lumen. This strongly supports that HcA uses neuroendocrine cells, mostly serotonin-producing cells, from intestinal crypts for its transport through the intestinal mucosa.

**Figure 5:**
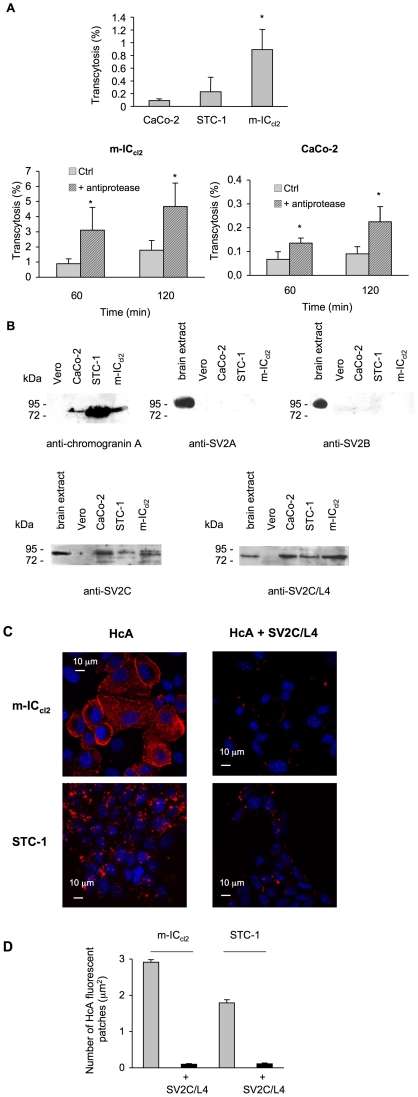
Transcytosis of BoNT/A through intestinal and neuroendocrine cell monolayers, and SV2C-dependent entry of HcA into cells. (**A**) Transcytosis of BoNT/A trough the intestinal neuroendocrine cell line STC-1, m-IC_cl2_, and Caco-2 cells. Cells were grown on filters (Transwell) until confluence. Integrity of cell monolayers was confirmed by ZO-1 labeling and non-permeability to FITC-labeled dextran (4300 Da). BoNT/A was added to the upper chamber and 60 min after incubation at 37°C, the toxin was assayed in the lower chamber by mouse bioassay. The results were expressed as the mean percentage (n = 9) of transcytosed BoNT/A corresponding to the ratio of mouse lethal dose 50 between the lower and upper well (inoculation titer). BoNT/A transport was significantly higher (*) in m-IC_cl2_ and in cells treated with anti-proteases (Student's t test). (**B**) Immunoblotting of cell lysates separated by SDS-PAGE with anti-chromograninA, anti-SV2A, anti-SV2B, anti-SV2C, and anti SV2C-L4 antibodies. (**C**) m-IC_cl2_ and STC-1 grown on glass cover slips were exposed to HcA-Cy3, alone or in combination with a 10-fold more molar concentration of SV2C/L4-GST for 10 min at 37°C. SV2C/L4 impaired HcA uptake in both m-IC_cl2_ and STC-1 cells. (**D**) The number of HcA fluorescent patches per µm^2^ in m-IC_cl2_ and STC-1 cells were reduced by 97% and 94% respectively (p<0.0001) by incubation with SV2C/L4. Each column represents the mean ± SEM (n = 250).

In addition, we checked whether BoNT/A can be transcytosed through the mouse neuroendocrine intestinal cell line STC-1 [45]. As shown in Figure 5A, the passage of biologically active BoNT/A was monitored from apical to basolateral side of STC-1 cell monolayers. The transcytotic passage of BoNT/A through STC-1 cells was not statistically different from that through Caco-2 enterocytes, but it was lower than through the mouse intestinal crypt cell line m-IC_cl2_ as shown in Figure 5A (p<0.05) and [30]. It is noteworthy that the passage yield through STC-1 cells was more difficult to assess (high standard deviation values), since these cells do not form tight junctions as epithelial cells. Since epithelial cells such as Caco-2 and HT29 cells express at the cell surface and secrete from apical and basolateral sides several types of proteases [46]–[48], we tested whether these proteases degrade BoNT/A, thus impairing or decreasing the transcytosis level. As shown in Figure 5A, a 2 to 4 fold higher level of BoNT/A transcytosis was observed in Caco-2 and m-IC_cl2_ cells incubated with a cocktail of anti-proteases. However, even in the presence of anti-proteases, BoNT/A transport was more efficient (20-fold) in m-IC_cl2_ than in Caco-2 cell monolayers. Thus, the decreased BoNT/A transcytosis through Caco-2 cells is not likely due to a higher protease degradation of BoNT/A before and/or after transport. STC-1 cells possibly also secrete proteases, and a higher level of BoNT/A transcytosis through this cell type might be expected. However, since STC-1 cells do not form tightly organized cell monolayers, the results of experiments with anti-proteases were inconclusive.

As we have previously found that m-IC_cl2_ express SV2C or an imunologically related protein as a putative BoNT/A receptor [30], we investigated the presence of SV2 proteins and chromogranin A in STC-1 and intestinal cells by Western blotting (Figure 5B). Chromogranin A was strongly expressed in STC-1 confirming its neuroendocrine type, and to a lower extent in m-IC_cl2_ and even less in Caco-2 cells, but not in Vero cells used as negative control. Antibodies against SV2A and SV2B showed no protein related to the expected size of SV2 (82 kDa) in intestinal and STC-1 cells. In contrast, specific SV2C antibodies directed against the N-terminal part or the intravesicular loop L4, which is assumed to be the receptor binding domain of BoNT/A [11], [14], recognized a protein with the expected size in the intestinal cells and STC-1, but not in Vero cells (Figure 5B). The specificity of SV2 antibodies is shown in Figure S1. Next, we investigated whether SV2C was involved in the entry of HcA into m-IC*_cl_*_2_ and STC-1 cells by a competition assay between fluorescent HcA and SV2C/L4. Cells grown on glass cover slides were exposed to HcA-Cy3 or a combination of fluorescent Hc with a 10-fold higher molar concentration of SV2C/L4 for 10 min at 37°C and then processed for microscopic observation. As shown in Figure 5C, HcA entered into m-IC*_cl_*_2_ and STC-1 cells, and SV2C-L4 greatly impaired the entry of HcA into both cell types by 97 and 94%, respectively, as determined by counting the number of fluorescent HcA patches per µm^2^ of cell area (Figure 5D), supporting the view that SV2C participates in the entry mechanism of HcA into cells.

### HcA targets specific neurons in the intestinal submucosa

**Figure 6:**
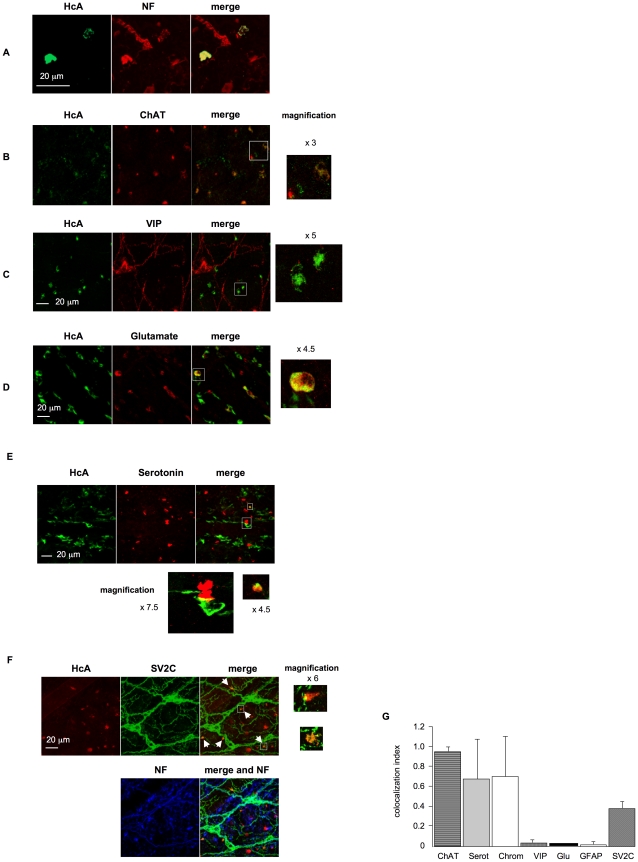
Neuronal cell types recognized by HcA in the intestinal submucosa after its inoculation into the intestinal lumen (30 min incubation). (**A**) Cells and neuronal structures labeled with HcA (green) were immunoreactive to anti-neurofilament (NF, red), indicating that HcA reached neuronal structures in the submucosa. Note that only few neurons and neuronal extensions were labeled with HcA (**B**) Co-staining with ChAT shows that some but not all ChAT-immunoreactive neurons were labeled with HcA. (**C**) Co-staining with anti-VIP antibodies showing no colocalisation with HcA. (**D**) Co-staining with anti-glutamate. Note that only a few neurons stained with anti-glutamate antibodies colocalized with HcA (scale bar = 20 µm). (**E**) Co-staining with anti-serotonin antibodies. Only few neurons stained with anti-serotonin antibodies colocalized with HcA. Note in the phase contrast in (**E**) that serotonin labeling was observed in some crypt intestinal cells and a few nerve endings in the submucosa. (**F**) Anti-SV2C and anti-neurofilament antibodies stained neurons and thin neuronal extensions in the submucosa. HcA labeled only certain nerve endings (arrows). Note that some SV2C-immunoreactive large cells with thicker extensions were neither stained with anti-neurofilament antibodies nor with HcA. (**G**) Quantification of colocalization between HcA and neurotransmitter markers in mouse intestinal submucosa. Results are expressed as colocalization index for which 1 represent 100% of colocalization between HcA and a neuronal marker. Colocalization index represents the ratio of the number of HcA positive terminal endings colocalized with one neurotransmitter marker to the total number of terminal endings labeled with HcA. Columns represent means ± SD accounting for at least 50 cells in three different experiments.

After 30–60 min incubation of fluorescent HcA into an intestinal loop lumen, HcA was detected in the intestinal submucosa, where it stained certain neuronal structures (Figure 6A and data not shown). Antibodies against neurofilaments allowed visualizing a complex and abundant network of neuronal cell bodies and neuronal extensions in the submucosal plexus immediately underneath intestinal villi and crypts. Only some of these neuronal structures were stained with HcA (Figure 6A).

BoNTs are well known to interact with cholinergic neurons and to specifically block spontaneous and evoked quantal acetylcholine release [4], [49]. However, it has been shown that BoNT/A and BoNT/E are also able to enter other neuronal cell types such as glutamatergic and gamma-aminobutyric acid (GABA)-ergic neurons, as well as astrocytes [50], [51]. Since ENS contains a large variety of neuronal cell types, we investigated the most representative types as putative targets of HcA in the mouse small intestine.

Cholinergic neurons were monitored by immunostaining with antibodies for choline acetyltransferase (ChAT). ChAT-immunoreactive neurons are abundant (about 55%) in the submucosal plexus, where they are involved in various gut functions including the control of evoked anion secretion by the jejunal and ileal epithelium, and they also interact with Peyer's patch follicles [52], [53]. Most of ChAT-immunoreactive nerve terminals from the intestinal submucosa were labeled with fluorescent HcA (Figure 6B and G).

Vasoactive intestinal peptide (VIP)-immunoreactive neurons are known to be located in jejunum and ileum submucosal plexus, as well as in other organs. VIP modulates several basic functions including blood flow, smooth muscle relaxation, and exocrine secretion [54]. VIP-immunoreactive neurons are estimated to represent about 45% of neurons from the submucosal plexus [53], [55]. Numerous cells were immunostained with anti-VIP antibodies in mouse intestinal submucosa, but a colocalization between VIP immunoreactivity and HcA staining was only observed in a few of them (less than 3%) (Figure 6C and G). Note that some of the filaments and cell bodies stained with anti-VIP antibodies did not colocalize with neurofilament staining, and may probably represent glial structures.

Glutamatergic neurons, which are the major neurons from the central nervous system involved in excitatory responses, are also present in ENS. Glutamate receptors have been detected in enteric neurons and glutamatergic enteric neurons where they have been found to mediate excitatory synaptic transmission, whereas only a subset of them are involved in sensory responses [56]. In mouse intestinal submucosa, only a low number of glutamatergic neurons (less than 1%), as evidenced by anti-glutamate antibodies, colocalized with HcA (Figure 6D and G).

Serotonin is also an important neurotransmitter in ENS, where it is involved in the control of motility, secretion and sensory functions. Serotonin is produced by 2 to 20% of all enteric neurons [33]. In our analysis only a few neuronal cell extensions were stained with anti-serotonin antibodies in the submucosal plexus, and some of them were also labeled with HcA, as shown in Figure 6E.

Glial cells were neither immunostained with anti-neurofilament antibodies nor with HcA, but exhibited a clear immunolabelling with GFAP antibodies (data not shown). These results support the view that HcA binding is mostly specific of nerve endings in the intestinal submucosa.

### HcA preferentially targets ChAT-immunoreactive neurons in mouse intestine musculosa

**Figure 7:**
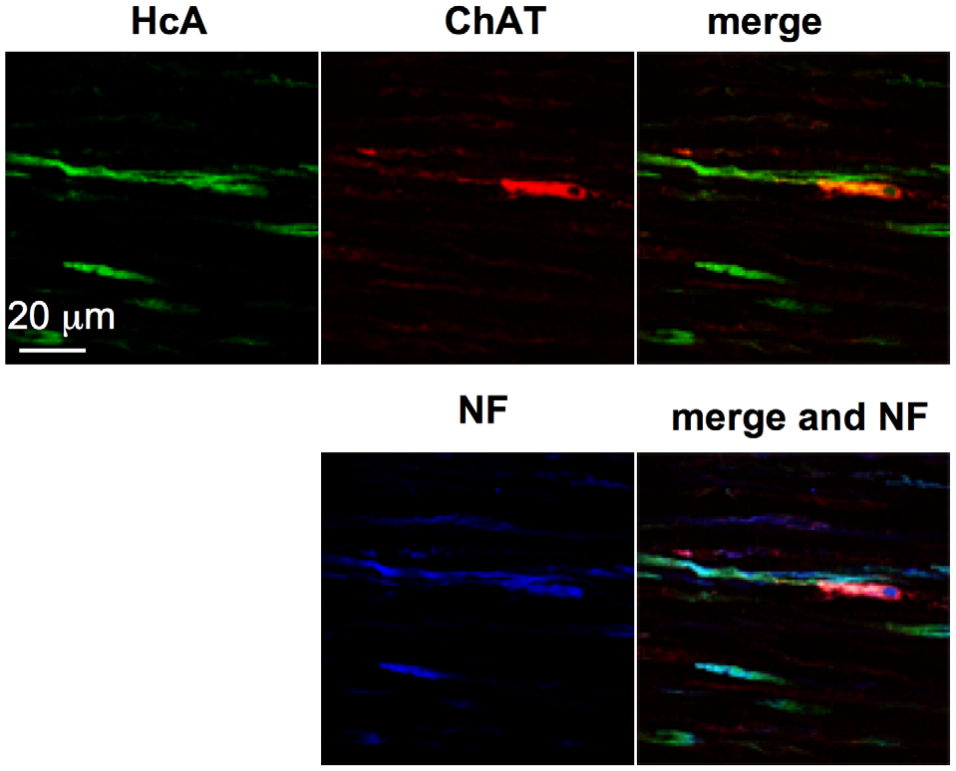
HcA labeled cholinergic neurons in the musculosa after 90 min exposure in the intestinal lumen. Co-staining with anti-ChAT and/or anti-neurofilament antibodies. Note that HcA preferentially stained neuronal cell extensions and to a lower extent cell bodies. (scale bar = 20 µm).

Also, we investigated the intestine musculosa, which is the predicted target tissue of BoNT/A for its inhibitory activity on intestinal motility. No significant HcA staining was observed in the musculosa 30 or 60 min after incubation with the fluorescent probe in the intestinal loop, and only a few cell bodies or extensions were stained after a longer incubation period (90–120 min) in our experimental conditions. Cell extensions stained with HcA colocalized with neurofilament staining (Figure 7). However, only some of the nerve endings were labeled with HcA. Almost all cell bodies labeled with HcA exhibited ChAT-immunoreactivity. Similar results of colocalization with ChAT-immunoreactive neurons were obtained using full length BoNT/A injected into the intestinal loop lumen and detected with anti-HcA antibodies (Figure S2). This is consistent with the fact that a large majority of neurons in the myenteric plexus are immunoreactive for ChAT, albeit many of them produce additional neurotransmitters [55]. No significant colocalization was observed between HcA staining and glutamate- or serotonin-producing neurons in the myenteric plexus (data not shown).

### BoNT/A receptor SV2 is expressed in certain intestinal neurons and glial cells

The protein receptor of BoNT/A on neuronal cells has been identified as SV2. Among the three SV2 isoforms, SV2C shows the highest affinity to BoNT/A *in vitro*, whereas BoNT/A binds to SV2A and SV2B with a lower strength [11], [14], [57]. SV2A is present in almost all neurons whatever their neurotransmitter type is, while SV2B shows a more restricted distribution. SV2C is reported to be present in a subset of neurons [58]. However, SV2 proteins are expressed not only in neuronal cells, but also in other cell types such as neuroendocrine cells, in particular in the gastrointestinal tract [59]. We investigated the distribution of SV2 proteins in mouse intestinal mucosa with our *in toto* tissue model. Numerous cell extensions in the submucosal plexus were stained with anti-SV2C antibodies (Figure 6F), whereas no specific staining was observed with anti-SV2B antibodies, and only a diffuse staining in certain crypt cells and cell extensions in the submucosa was evidenced with anti-SV2A antibodies (data not shown). The mouse intestinal crypt cell line m-IC_cl2_ was found to express SV2C at a higher level than in enterocyte-type cell lines (Figure 5B and [30]). However, in our *ex vivo* conditions intestinal crypt cells were not, or only weakly immunoreactive with anti SV2C antibodies. This does not preclude that a subset of crypt cells express a significant level of SV2C that was not detected in our conditions. Most of SV2C-immunoreative filaments in the submucosa, were also labeled with anti-neurofilament antibodies, indicating that SV2C is widely distributed in neuronal cells and neuronal extensions from the small intestine. However, HcA signal was observed in only some neuronal endings bearing SV2C, but not along the neuronal extensions stained with anti-SV2C antibodies (Figure 6F). Only a low proportion of SV2C-immunoreactive cells were labeled with HcA (Figure 6G). It is worth noting that a distinct network of thin filaments around small blood vessels was stained with anti-SV2C, and only weakly with anti-neurofilament antibodies. No specific binding of HcA was observed on these structures (Figure S3A and B). In the intestinal submucosa and musculosa, large cells with wide cellular bodies and short extensions were stained with anti-SV2C antibodies, but not with anti-neurofilament antibodies. Co-labelling with anti-GFAP indicated that they were glial cells (Figure S3C). However, HcA, as reported above, was not found to label glial cells.

## Discussion

The standard scheme of botulism intoxication includes BoNT transit through the digestive tract, passage across the intestinal epithelial barrier and subsequent delivery to the blood circulation and dissemination to the target motor nerve endings. Indeed, BoNT has been found to be absorbed preferentially from the upper small intestine, but also from the stomach in experimental rodents, and to be delivered in the blood and lymph circulation [21], [23], [24], [60]. The aim of this study was to identify the entry pathway and target cells of BoNT/A in the mouse intestinal wall. For that, we checked the activity of BoNT/A in mouse intestine following intralumenal administration and we used the fluorescent Hc domain, which is the functional binding domain of BoNT, to monitor the trafficking of BoNT/A.

First, we tested whether BoNT/A injected into intestinal lumen was able to enter intestinal mucosa and to induce local effects on the intestine. *In vitro* studies have already shown that BoNT/A reduces cholinergic transmission in gastrointestinal smooth muscles as well as pylori and Oddi sphincter muscles by inhibiting ACh release [40], [41], [61]–[63]. In our experimental conditions, BoNT/A passed through the epithelial intestinal barrier and by diffusion through the extracellular space, locally targeted intestinal neurons independently of the blood circulation. BoNT/A reduced the frequency of spontaneous contractions of small intestine and inhibited the contractile response evoked by electric field stimulation within 2–4 h after intralumenal administration. Since carbachol was still able to stimulate muscle contraction after BoNT/A treatment, this supports a toxin-dependent inhibition of ACh release. To the best of our knowledge, this is the first report demonstrating a local intestinal effect of botulism after intralumenal administration of purified BoNT/A. Constipation is often (about 70%), but not always, associated with food-borne botulism and its participation to the progression of the disease is unknown [64], [65]. However, constipation is a major and early symptom of botulism resulting from an intestinal colonization by *C. botulinum* such as during infant botulism [66], [67]. This digestive symptom might result from a local effect of BoNT after crossing the intestinal barrier instead of toxin dissemination through the general circulation. BoNT locally synthesized in the intestine is possibly absorbed in a higher local concentration able to induce an intestinal muscle paralysis, than toxin orally ingested which disseminates more broadly through the digestive tract. Interestingly, BoNT/A-dependent inhibition of evoked smooth muscle contraction was significantly prevented by preincubation with the intravesicular domain of SV2C. This indicates that a protein related to SV2C/L4 might impair the BoNT/A passage through the intestinal barrier and/or toxin uptake by the underlying nerve terminals. We have previously found that SV2C, or a related protein, is part of BoNT/A receptor mediating toxin transcytosis through cultured intestinal cell monolayers [68]. In addition, SV2C/L4, which is expressed by intestinal and neuroendocrine STC-1 cells (Figure 5B), significantly prevented HcA entry into the intestinal crypt m-IC_cl2_ and STC-1 cells (Figure 5C, D). Taken together, these data suggest that SV2C, or a related protein, facilitates BoNT/A uptake through the mouse intestinal barrier.

BoNT/A trafficking in mouse intestinal wall was investigated with fluorescent HcA as already used [38]. First, we investigated the potential binding sites of HcA by using mouse small intestine cryosections overlaid with fluorescent probes. Thereby, HcA was found to bind only to certain cell types of the intestinal mucosa, preferentially from intestinal crypts, whereas enterocytes showed only a weak staining of the brush border. In contrast, it was reported that the botulinum complexes type A or type C (BoNT and associated non-toxin proteins, ANTPs), strongly bind to the epithelia cell surface and goblet cells of guinea pig small intestine. Moreover, this binding was shown to be mediated by the hemagglutinins HA1 and HA3b, which interact with distinct gangliosides and/or glycoproteins [24], [29], [60], [69] from those recognized by the neurotoxin alone [11], [14]. This certainly accounts for the differential binding between progenitor toxin and BoNT to intestinal epithelial cells.

The functions of ANTPs are still controversial. Ancillary proteins probably participate in BoNT protection from degradation inside the digestive tract in a dose-dependent manner, particularly in the stomach [21]. In addition, it is assumed that HAs are involved in the internalization of progenitor type C toxin into intestinal cells and subsequently in the small intestine [24], [29], [70], and that they disrupt the intestinal epithelial barrier facilitating toxin absorption via the paracellular route [71]–[74]. However, since BoNT/A absorption from mouse stomach or small intestine was found to occur independently of the presence of ANTPs, HAs might have not an essential role but an additional facilitating effect on BoNT/A passage through the intestinal barrier. At pH around neutrality, as found in the intestine, BoNT dissociates from ANTPs [75], and thus might be absorbed through the epithelial intestinal barrier independently of ancillary proteins. In our model of ligated mouse intestinal loops and recording of muscle contraction, BoNT/A entered the intestinal mucosa in the absence of ANTPs supporting that ANTPs are not absolutely required in the intestinal uptake of BoNT/A.

The main finding of the *ex vivo* intestinal loop experiments was that fluorescent HcA preferentially recognized certain crypt cells. Intestinal crypts contain stem cells which proliferate and differentiate in four main cell types including enterocytes, mucus, endocrine, and Paneth cells, which are the most abundant cell type at the bottom of intestinal crypts [76]. HcA labeling was observed in chromogranin- and serotonin-immunoreactive cells, but not in Paneth cells. It is noteworthy that HcA was detected in only some, but not all, chromogranin-immunreactive cells. This strongly suggests that HcA specifically binds to a subset of neuroendocrine cells from intestinal crypts, which possibly represent the preferential site of toxin uptake.

BoNT/A has been found to pass through intestinal cell monolayers grown on filter by a transcytotic mechanism [26], [27]. We have previously reported that the passage rate of biologically active BoNT/A was higher (about 10-fold more) through the mouse crypt cell line m-IC_cl2_ than through the colonic enterocyte lines Caco-2 and T84 [30]. Here, we found that the neuroendocrine intestinal cell line STC-1 also permits a transcytotic passage of BoNT/A not statistically different from that through Caco-2 cells and to a lower extent than in m-IC_cl2_ cells. This further supports that a subset of intestinal crypt cells constitute a preferential entry site of BoNT/A within the intestinal mucosa. However, STC-1 cells, which derive from an intestinal endocrine tumor, secrete several hormones and neuropeptides, but not serotonin [45]. Thereby, STC1 cells are not the most representative cells of the serotonin-reactive cells co-labeled with HcA, which have been identified in intestinal loop tests. To the best of our knowledge, no intestinal serotonin-secreting endocrine cell line is known at present. m-IC_cl2_ cells, which have been isolated from fetal mouse intestine, express various markers of intestinal cells and thus are a totipotent intestinal crypt cell line in a less differentiated state than the tumor cell line STC-1 [77]. m-IC_cl2_ cells show a neuroendocrine-like phenotype since they synthesize chromogranin A albeit to a lower extent than STC-1, but more significantly than Caco-2 cells (Figure 5B). In addition, m-IC_cl2_ cells retain transport pathways similar to those of native crypt cells. Indeed, in contrast to colonocytes such as Caco-2 and HT29 cells, m-IC_cl2_ cells express polymeric Ig receptors (pIgRs), which mediate the transepithelial transport of polymeric IgA and IgM [77]. It is noteworthy that only a low number of cells (1 to 2) from each intestinal crypt were labeled by HcA and are likely susceptible to transport the toxin through the intestinal barrier. This might account for the low rate of BoNT absorption from the digestive tract to the general circulation. Indeed, the transport rate of BoNT/B from rat duodenum to the lymphatic circulation has been estimated from 0.01 to 0.1% [22]. In addition, chromogranin-immunoreactive cells are distributed throughout the gastrointestinal tract, but are more predominant in the pylorus and duodenum [78]. This might support the observation that the upper small intestine is the preferential site of BoNT absorption [22]–[25].

Using the intestinal loop model, we found that neuronal cells in the submucosa and more lately in the musculosa were stained with HcA. However, not all the neuronal cells were recognized by HcA as tested by co-labeling with antibodies against neurofilament, indicating that HcA targeted specific neurons in the intestine. About half of neurons in the submucosa are ChAT-immunoreactive, and most of them produce other neurotransmitter types [53], [55]. As previously found [79], HcA stained most of the cholinergic neurons (more than 90%) of the submucosa. However, in contrast to previous findings [79], BoNT/A recognized other neuronal cells in the intestinal mucosa, albeit to a lower extent. Indeed, a low proportion of VIP-immunoreactive neurons, which are also largely present in the submucosa (about 45%) [53] as well as a low proportion of glutamatergic and serotoninergic neurons were targeted by HcA. It is noteworthy that the BoNT/A receptor, assigned to ganglioside GD_1b_/GT_1b_ and SV2 protein [6], [7], [10], [14], is part of a synaptic vesicle complex, which contains additional membrane proteins including vesicular glutamate transporters (cGLY-1 and vGLUT-2) [80], supporting that BoNT/A may target glutamatergic neurons. BoNTs are known to block the release of ACh, but also that of other neurotransmitters and neuropeptides the effects of which are still poorly known [61], [81]–[86]. The BoNT/A-dependent inhibitory activity on non-ChAT neurons in the intestine may contribute to the local effects of the toxin.

In the myenteric plexus, HcA stained essentially cholinergic neurons, which are the predominant neuronal cells in this tissue (about 80%) [53]. But, only a part of ChAT-immunoreactive neurons were labeled with HcA. This might be due either to a low number of Hc molecules reaching the myenteric plexus, or to the fact that HcA recognized only a subset of ChAT-reactive neurons, which remains to be determined. Targeting of cholinergic neurons of the myenteric plexus by HcA is consistent with constipation which frequently occurs during botulism, and with the BoNT/A-dependent inhibition of evoked intestinal contractions in the *ex vivo* experiments.

SV2C, the protein receptor with the highest affinity to BoNT/A [10], [14], was the main isoform detected in the mouse intestine, whereas no, or a weak staining was obtained with anti-SV2B or -SV2A antibodies. Immunostaining with anti-SV2C antibodies was observed in cells with thin arborescent extensions, which were also labeled with anti-neurofilament antibodies. However, SV2C staining did not match all the cells labeled with anti-neurofilament antibodies, indicating a SV2C distribution in a broader range of cell types than neuronal cells in the intestinal mucosa. HcA did not bind all along the neuronal extensions or cell structures which are recognized by anti-SV2C antibodies, but only in discrete zones. One possibility is that HcA staining was too weak to be visualized. Alternatively, the BoNT/A high-affinity receptors, which consist of a ganglioside part and a protein part such as SV2C [11], [14]–[17], are distributed in only some restricted areas on the neuronal cell surface. This is supported by the observed colocalization between HcA and SV2C, which is restricted to only some cell areas (Figure 6F). Moreover, SV2, which is an integral protein of the synaptic vesicle membrane, has to be exposed to the extracellular compartment to be accessible to BoNT/A, as during synaptic vesicle fusion with the presynaptic membrane. Another type of thin cell extensions stained with anti-SV2C antibodies, formed a dense network all around microvessels (Figure S2). These neuronal extensions, which were only faintly stained with anti-neurofilament antibodies, are probably involved in vessel innervation [87], but were not observed to be targeted by HcA under our experimental conditions. In addition, glial cells identified by GFAP immunofluorescence were also stained with anti-SV2C antibodies but not with HcA (Figure S3C). Thereby, SV2C seems to have a broad distribution and to form BoNT/A high-affinity receptor only when associated with gangliosides in certain neuronal membrane domains.

When passed across the intestinal barrier, BoNT is delivered to the connective tissue of the submucosa, where the toxin can have access to microvessel endothelial cells. BoNT passage into the blood and lymph circulation, supposes a transcytotic transport of the toxin through the endothelial cell barrier of vessels. In ligated intestinal loop experiments, no staining of endothelial cells forming small vessels or microvessel structure was observed in the submucosa and musculosa. Under natural conditions, BoNT binding to endothelial cells is possibly low and/or transient permitting the passage of the toxin into the vessel lumen. In our experimental conditions, the blood circulation was interrupted, thus preventing HcA passage into vessels. But, the diffusion through the matrix of connective tissue can mediate the toxin trafficking until the target cells in the submucosa and musculosa. Indeed, HcA staining of neuronal cells in the submucosa after 30–60 min incubation of the probe into the intestinal lumen and later (60–120 min) in the musculosa, as well as the decrease in fluorescence intensity of HcA from the mucosa to musculosa likely reflects the progressive diffusion of the recombinant protein in the tissue extracellular matrix. It cannot be ruled out that a shorter time period may be required in natural conditions for the passage of low amounts of HcA across the intestinal epithelial cell barrier and subsequent targeting to neuronal cells. Alternatively, another mode of HcA transport might be involved. Indeed, like TeNT, which undergoes a retrograde transport into motor neurons, a transcytotic mechanism might also be supported by neuronal cells from the ENS to disseminate BoNT/A to target neuronal cells both locally and even at distance from the intestine. One possibility is that BoNT/A uses non-cholinergic neurons such as those identified with fluorescent HcA to be transported to other target neurons. Indeed, neurons from ENS are highly interconnected between them and with neurons of the central nervous system [31], [32]. Moreover, it has been recently found that BoNT/A can use a retrograde transport and transcytosis to migrate from peripheral neurons to central circuits [88], [89]. This opens novel ways of investigation to unravel the mechanism of BoNT transport from the intestinal barrier to target motoneurons, which is still poorly known.

In conclusion, in this work we have shown that BoNT/A enters the intestinal mucosa, possibly via an uptake process involving SV2C or a related protein, and impairs spontaneous and electrically-evoked muscle contractions. Following intralumenal administration, HcA preferentially recognized a subset of neuroendocrine crypt cells in the mouse upper small intestine, which likely represents the main pathway for toxin entry and passage across the epithelial cell barrier. HcA diffused progressively within the submucosa, and later reached the musculosa, where it targeted specifically some cholinergic neurons and to a lower extent glutamatergic and serotoninergic neurons. The implication of these non-cholinergic neurons in the botulinum intoxication process remains to be determined.

## Materials and Methods

### Ethic statements

All experiments were performed in accordance with French and European Community guidelines for laboratory animal handling. The protocols of experiments were approved by the Pasteur Institute (Agreement of laboratory animal use n° 75-279).

### Reagents

The primary antibodies used recognized neurofilament (Sigma; mouse, 1∶500 dilution), Glial Fibrillary Acidic Protein (GFAP) (Sigma; rabbit, 1∶200 dilution), Choline Acetyltransferase (ChAT) (Chemicon International, Temecula, CA, USA; goat, 1∶100 dilution), VIP (Abcam; rabbit, diluted 1∶200), Serotonin (Sigma; rabbit, diluted 1∶100; and Abcam ; goat, 1/200 dilution), Glutamate (Chemicon; rabbit, diluted 1∶200), SV2A, SV2B (Synaptic systems; rabbit, diluted 1∶200), SV2C (Santa Cruz; goat, diluted 1∶200), chromogranin A (Abcam; rabbit, diluted 1∶200). For Western blotting rabbit anti-SV2C (Abcam 33892) and rabbit anti-SV2C intravesicular domain were used. SV2C/L4 domain (amino acid 454 to 579) was produced as a GST-fusion protein [30] and was used to immunize rabbits. Specific antibodies against SV2C/L4 were purified by immunoaffinity with SV2C/L4 produced as a histidine-tagged protein in pET28 vector (Novagen) and immobilized on cyanogen bromide activated Sepharose-4B (GE Healthcare).

The secondary antibodies used were: Cy5 coupled donkey anti-mouse IgG (US Biological), Cy3 coupled donkey anti-goat IgG (US Biological), Alexa488 donkey coupled anti-goat IgG (Invitrogen), Alexa488 coupled anti-rabbit IgG (Invitrogen). 4′-6′-Diamidino-2-phenylindole (DAPI, SIGMA) was incubated with secondary antibodies to stain cell nuclei.

### Animals

Adult male IOPS mice (20–25 g body weight) purchased from Charles River Laboratories (L'Arbresle, France) were anesthetized by inhalation with Isoflurane (AErrane, Baxter S.A., Lessines, Belgium), and euthanized by dislocation of the cervical vertebrae, as specified by the CNRS Animal Ethics User's Committee.

### Protein production

BoNT/A was produced and purified as previously described [90].

Recombinant His-tag Hc fragment of BoNT/A was produced and purified from pET28b vector containing DNA encoding for HcA cloned into *BamHI* and *SalI* sites, as previously described [91]. A recombinant derivative plasmid encoding HcA with a N-terminal tag containing 4 cystein residues, as described for TeNT-Hc [92], was performed inserting the two complementary oligonucleotides P1132 (5′-TATGGCAGAGGCAGCAGCACGAGAGGCTTGTTGTCGAGAGTGTTGTGCACGAG-3′) and P1131 (5′-GATCCCTCGTGCACAACACTCTCGACAACAAGCCTCTCGTGCTGC TGCCTCTGCCA-3′) into *NdeI* and *BamHI* sites of pET28b vector. HcA with 4 Cys tag was then labeled with either maleimide Alexa 488 reagent (InVitrogen, Cergy-Pontoise, France) or maleimide Cy3 (Amersham, Les Ulis, France) according to the manufacturer's recommendations.

cDNA coding the intraluminal SV2C fragment L4 (amino acid 454 to 579) was PCR-amplified as previously described [68] and cloned into pGEX-2T. The fusion protein, GST-SV2C/L4, was produced in *E. coli* BL21 and purified on glutathione agarose matrix (SIGMA) equilibrated with 50 mM Tris-HCl, 300 mM NaCl, pH 7.5 and eluted with 40 mM reduced glutathione in the same buffer. Purified fusion protein was then dialyzed against phosphate balanced solution (PBS) before use.

### Tension recordings on isolated mouse ileum segments

The mouse ileum was removed and placed in an oxygenated standard Krebs-Ringer solution composed of 154 mM NaCl, 5 mM KCl, 2 mM CaCl2, 1 mM MgCl2, 11 mM glucose and 5 mM HEPES (pH 7.4). An ileum segment of about 2 cm length was extensively washed to flush out the intestinal content, and mounted in a silicone-lined chamber (4 ml volume), bathed in the standard medium. For tension measurements, one of the ends of the ileum segment was tied with silk thread, via an adjustable stainless-steel hook, to an FT03 isometric transducer (Grass Instruments, AstroMed, W. Warwick, RI, USA), and the other end was ligatured and pinned onto the silicone-coated bath via stainless-steel micro pins. BoNT/A (5 µg/ml) diluted in the Krebs-Ringer solution was injected into the ileum segment, between the two ligatures, with a micro syringe. Electric field stimulation was performed with an electrode assembly, placed along and on both sides of the length of the ileum segment, and connected to S-48 Grass stimulator. Preparations were usually stimulated with pulses of 0.15 ms duration at 30 Hz for 15 or 25 s, every hour. The resting tension was adjusted for each preparation investigated with a mobile micrometer stage (to allow incremental adjustments of ileum length) in order to obtain maximal spontaneous or evoked contractile responses, and was monitored during the whole duration of the experiment. Carbachol (Sigma, 20 µM) was added to the bath solution of ileal segment exposed to BoNT/A for 3–4 hours to check that ACh receptors were not affected by intoxication. Tension signals from the isometric transducer were amplified, collected, and digitized with the aid of a computer equipped with a DT2821 analogue to digital interface board (Data Translation, Marlboro, USA) and expressed in g or N. Data acquisition and analysis were performed with a program kindly provided by Dr. John Dempster (University of Strathclyde, Scotland). All experiments were performed at 22±0.5°C.

### *Ex-vivo* experiments with mouse ligated ileal loops

Segments of mouse ileum (2 cm length) were removed and immersed in oxygenated Krebs-Ringer solution. After extensive wash to flush out the luminal contents, segments were ligated on both extremities and injected with a micro syringe with fluorescent HcA (0.5 µg) diluted in 150 µl of Krebs-Ringer solution (5.5 10^−8^ M). The injection site was isolated with another ligature and ileal segments were incubated in oxygenated Krebs-Ringer solution for different times (30 to 120 min) before washing. Tissues were cut along the mesenteric border and pinned out with the mucosal surface facing down in a silicone-lined Petri dish. After fixation with 4% paraformaldehyde (PFA; 1 h, 22°C), tissues were washed in phosphate buffer saline (PBS) and autofluorescence quenched with 50 mM NH_4_Cl (30 min, 22°C).

### Microdissection and indirect immunofluorescence of dissected ileal loops

Specimens were dissected by carefully separating the mucosa and submucosa from the muscle layers under a microscope. Whole-mount preparations of the myenteric and submucosal plexuses of the ileum were permeabilized and blocked with PBS/bovine serum albumin (BSA) 2%/Donkey Serum 10%/Triton 2% for 1 h at room temperature (RT) and incubated with primary antibody for 16 h at RT. After 3 washes of 10 min in PBS, tissue sections were incubated for 4 h at RT with the appropriate secondary antibodies diluted 1∶500 in PBS/BSA 2%/Triton 2%. After being washed in PBS (3×10 min), tissues were mounted in Mowiol (Polysciences Europe, Eppelheim, Germany) and analyzed using a Zeiss confocal laser scanning microscope and a ×63 oil immersion objective (N.A. 1.4). For quantitative analysis of co-localization of HcA with other markers, images of 512×512 pixels were taken from series of optical sections of 0.8 µm thickness. The colocalisation was analyzed with the Zeiss LSM Image Browser software, on 100 HcA-immunoreactive varicosities for each marker using the merged image from the different experiments. For analysis of BoNT/A internalization into intestinal cells, total fluorescence intensity was quantified in serotonin-positive cells in at least 40 optical fields taken from three independent experiments. Data are presented as the mean ± SD.

### Small intestine cryosections

Segments of the ileum were removed and immersed in oxygenated Krebs-Ringer solution. After extensive wash to flush out the luminal contents, the tissues were embedded in OCT embedding medium (Tissue-Tek, Miles Laboratories, Naperille, IL, USA), and stored at −80°C. Sections (5 µm) were cut with a cryostat-microtome and thaw, mounted onto SuperFrost glass slides (Fisher Scientific, Illkirch, France). For HcA binding experiments, sections were removed from the freezer and incubated for 30 min with 10 µg/ml Alexa- or Cy3-labeled HcA in PBS/BSA (1%). After 3 washes in PBS, tissue sections were fixed with 4% PFA for 20 min at RT (22°C), rinsed in PBS and autofluorescence was quenched with 50 mM NH_4_Cl (15 min, 22°C). After permeabilization with 1% triton X-100 in PBS for 10 min, tissues were stained with TRITC-phalloidin (Sigma, 0.4 µg/ml), TRITC-*Urex europaeus* agglutinin type 1 (UEA1, 1 µg/ml), immunostained with anti-neurofilament 200 (Sigma; mouse, diluted 1∶500) and Cy3 coupled goat anti-mouse IgG (Sigma, diluted 1∶250). After 3 washes in PBS, sections were mounted in Mowiol and observed with a Zeiss confocal laser scanning microscope and a ×25 objective. For quantitative analysis of co-localization of HcA with markers, images of 512×512 pixels were taken from serial optical sections of 1 µm thickness. Data are presented as the mean ± SD.

### BoNT/A transcytosis and immunofluorescence experiments

The mouse neuroendocrine intestinal cell line STC-1 [45], m-IC_cl2_ [77] and Caco-2 (human colon) cells were grown on filter (Transwell, Corning) in Dulbecco's modified Eagle's medium (DMEM, Invitrogen) supplemented with 10% fetal calf serum (FCS, Invitrogen) until confluence. Integrity of tight junctions was confirmed by ZO-1 labeling and non-permeability to FITC-labeled dextran (4300Da, Sigma-Aldrich) (data not shown). BoNT/A as prepared previously [30] was added to the apical chamber. After 60 min incubation at 37°C, medium from the basal chamber was collected and BoNT/A was assayed by the mouse bioassay as previously described [30]. In the experiments with anti-proteases, culture medium in the apical and basolateral chambers was replaced with Dulbecco's medium containing 1% bovine serum albumin (BSA) and 1× anti-protease coktail without EDTA (Calbiochem) 30 min prior addition of BoNT/A into the apical compartment. BoNT/A transcytosis was monitored in basolateral medium samples after 60 and 120 min incubation at 37°C by a biological assay as previously described [30].

m-IC_cl2_ and STC-1 cells grown on glass coverslips (coated with poly-ornithine, Invritrogen, for STC-1 cells) were exposed to HcA-Cy3 (2.5 µg/ml), alone or in combination with a 10-fold more molar concentration of SV2C/L4-GST for 10 min at 37°C. Cells were washed twice with PBS, fixed with 4% PFA and mounted in Mowiol. The number of HcA fluorescent patches per µm^2^ was evaluated in m-IC_cl2_ and STC-1 cells by counting in images of 512×512 pixels (10 optical fields), taken from serial optical sections of 1 µm thickness from 3 experiments. Data was evaluated using ImageJ software (http://rsbweb.nih.gov/ij/).

### SDS-PAGE and immunoblotting

Cells were lysed with boiling Laemmli buffer and lysates were homogenized by passages through a 26-gauge needle. An equal amount of protein (100 µg) from each boiled sample was loaded on a SDS-polyacrylamide (10%) gel. Samples separated by SDS-PAGE were transferred to nitrocellulose membrane (Amersham) and blocked in phosphate saline buffer containing 5% dried milk, washed with Tris-buffered saline containing 0.1% Tween20 (TBST), incubated with primary antibodies diluted in TBST overnight at room temperature, then washed 5 times for 10 min, and incubated for 1 h at room temperature with HRP-protein A diluted in TBST. After membrane washing in TBST, the specific signal was detected by enhanced chemiluminescence.

Specificity of the anti-SV2A antibodies was tested using lysate from SV2A-transfected cells (Santa Cruz). The lysate (10 µg) was run on a 10% SDS-PAGE, transferred on nitrocellulose, and blotted with anti-SV2A, anti-SV2B, anti-SV2C, and anti-SV2C-L4 antibodies. The specificity of anti-SV2B and anti-SV2C antibodies was tested on rat brain extract by competition with peptides, which had been used for immunization. Rat brain extract (5 µg, Santa Cruz) was run on a 10% SDS-PAGE, transferred on nitrocellulose, and blotted with anti-SV2B or anti-SV2C antibodies preincubated or not with the indicated SV2B or SV2C peptides (10 µg, Synaptic System) for 30 min at room temperature.

### Statistics

Values in the text are expressed as the mean ± SD, unless otherwise indicated. Differences between means were tested using Student's *t*-test, and *p*-values<0.05 were taken to indicate significance.

## Supporting Information

Figure S1 Specificity of the anti-SV2 antibodies tested by Western blot. (**A**) In lysate of cells overexpressing SV2A, the corresponding 75–80 kDa band was detected with anti-SV2A, but not with anti-SV2B, anti-SV2C, and anti-SV2C-L4 antibodies. (**B**) In rat brain extract, a band corresponding to SV2B was detected with anti-SV2B antibodies alone or pre-incubated with SV2C peptide. No band was observed when anti-SV2B antibodies were preincubated with SV2B peptide. Conversely, a band corresponding to SV2C was detected in rat brain lysate with anti-SV2C antibodies alone or pre-incubated with SV2B, but to a much lower extent when antibodies were pre-incubated with SV2C peptide.
(TIF)

Figure S2 Immuno-detection of BoNT/A (10^5^ LD_50_/ml) injected into the lumen of a ligated ileal segment (4 h incubation) using antibodies against HcA. BoNT/A (green) was detected in filament structures of the musculosa co-labeled with anti-NF (blue) and anti-ChAT (red), showing that BoNT/A, injected in the intestinal lumen, reached cholinergic nerve endings in the musculosa. (scale bar = 20 µm).
(TIF)

Figure S3 Immunolabeling pattern of SV2C in intestinal submucosa. (**A**) HcA (red) was injected into the lumen of a ligated intestinal loop and incubated for 1 h at room temperature. A network of thin cell extensions around microvessels in the submucosa were stained with anti-SV2C (green), and not with HcA. HcA only decorated some filament extremities. (**B**) Thin neuronal extensions around microvessels were labeled with anti-neurofilament (NF, blue) and anti-SV2C (red) antibodies. (C) Glial cells in the submucosa were stained with anti-GFAP (green) and anti-SV2C (red), but not with HcA (not shown) (scale bar = 20 µm).
(TIF)
