# Supplementary figures and images for: Preferential Entry of Botulinum Neurotoxin A Hc Domain through Intestinal Crypt Cells and Targeting to Cholinergic Neurons of the Mouse Intestine
Source: PLoS Pathog. 2012 Mar 15;8(3):e1002583. doi: 10.1371/journal.ppat.1002583 (PMC3305446; doi:10.1371/journal.ppat.1002583)

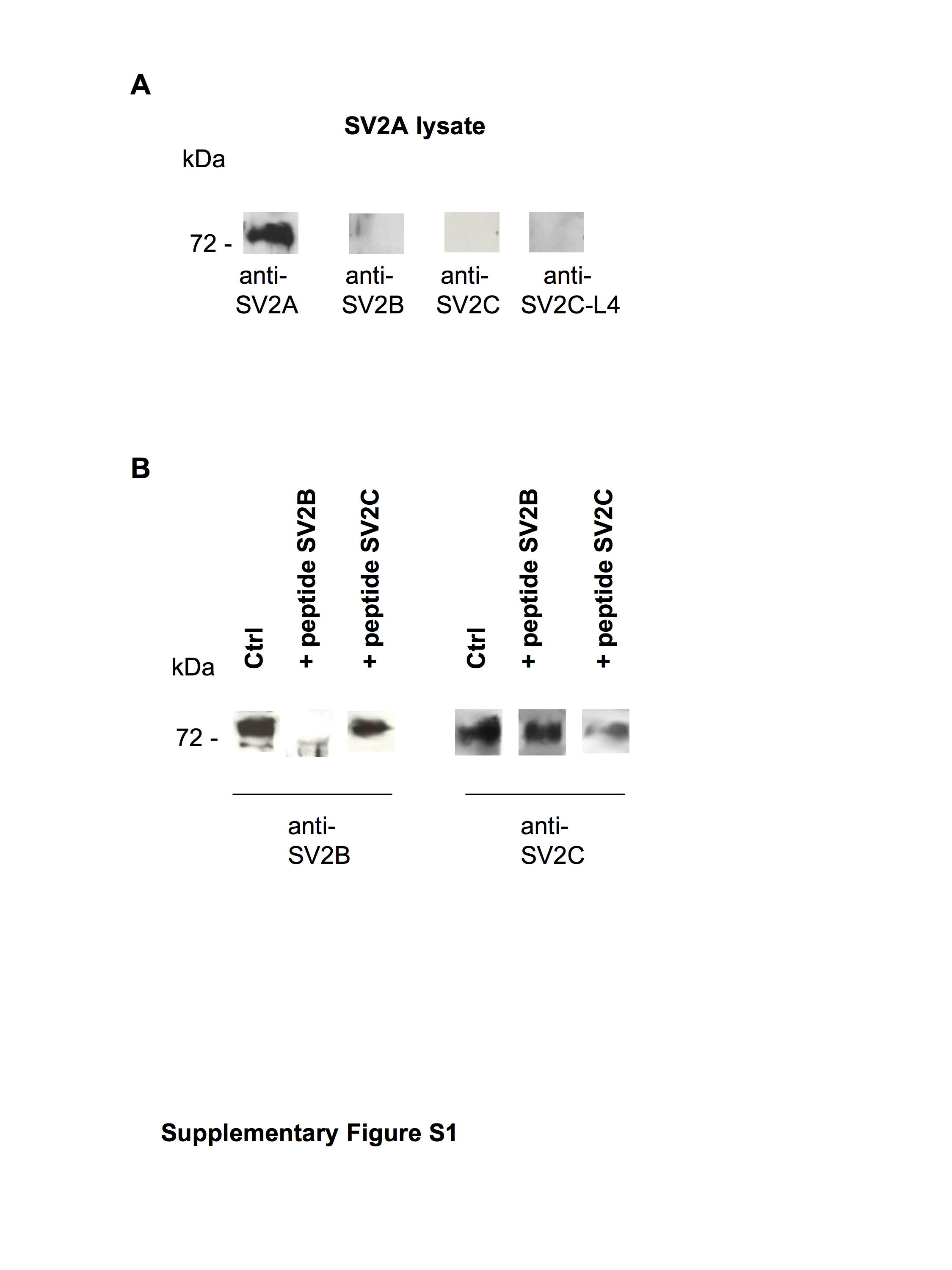

Supplement: Figure S1 — Specificity of the anti-SV2 antibodies tested by Western blot. (A) In lysate of cells overexpressing SV2A, the corresponding 75–80 kDa band was detected with anti-SV2A, but not with anti-SV2B, anti-SV2C, and anti-SV2C-L4 antibodies. (B) In rat brain extract, a band corresponding to SV2B was detected with anti-SV2B antibodies alone or pre-incubated with SV2C peptide. No band was observed when anti-SV2B antibodies were preincubated with SV2B peptide. Conversely, a band corresponding to SV2C was detected in rat brain lysate with anti-SV2C antibodies alone or pre-incubated with SV2B, but to a much lower extent when antibodies were pre-incubated with SV2C peptide. (TIF) [file ppat.1002583.s001.tif]

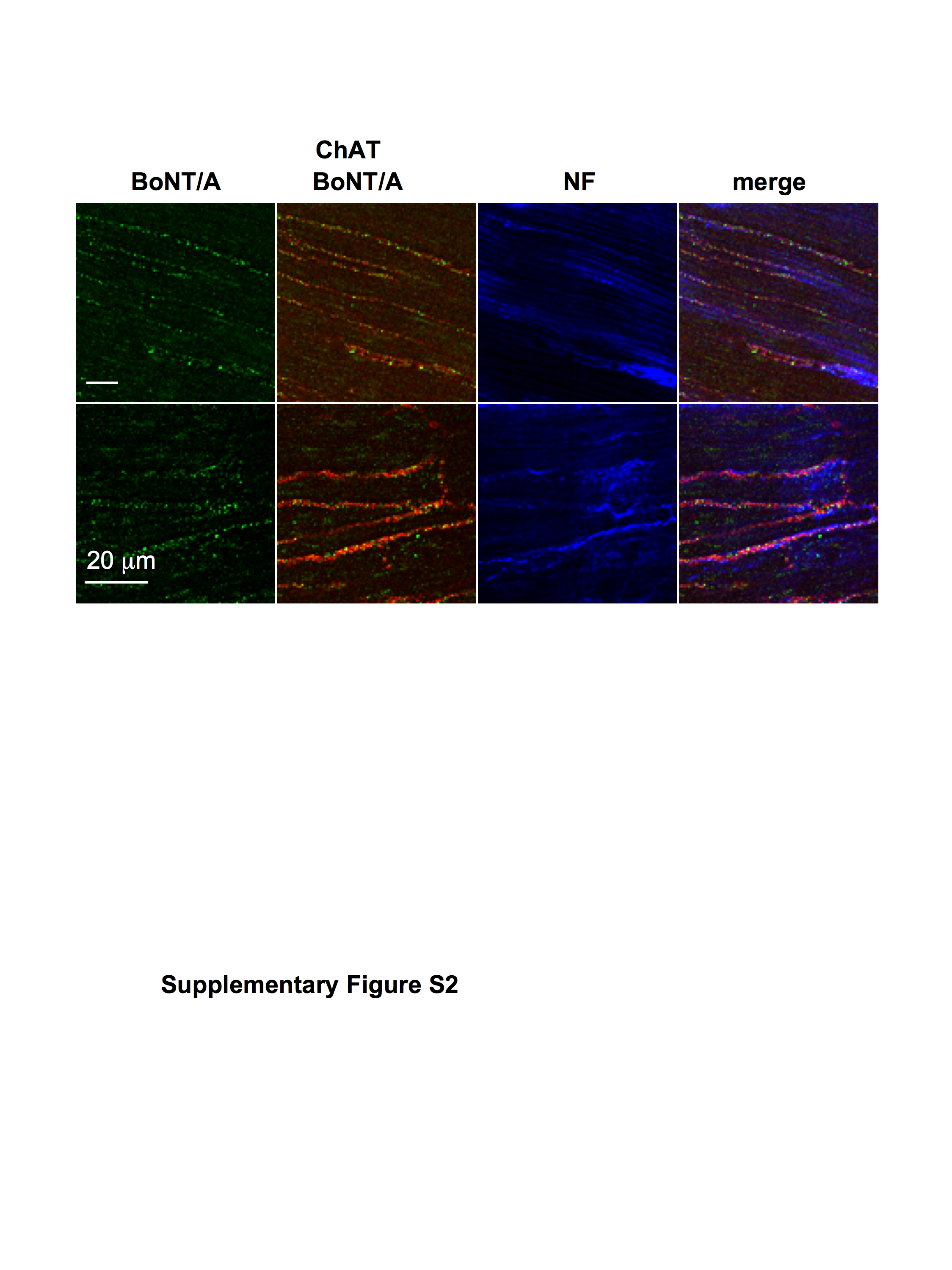

Supplement: Figure S2 — Immuno-detection of BoNT/A (105 LD50/ml) injected into the lumen of a ligated ileal segment (4 h incubation) using antibodies against HcA. BoNT/A (green) was detected in filament structures of the musculosa co-labeled with anti-NF (blue) and anti-ChAT (red), showing that BoNT/A, injected in the intestinal lumen, reached cholinergic nerve endings in the musculosa. (scale bar = 20 µm). (TIF) [file ppat.1002583.s002.tif]

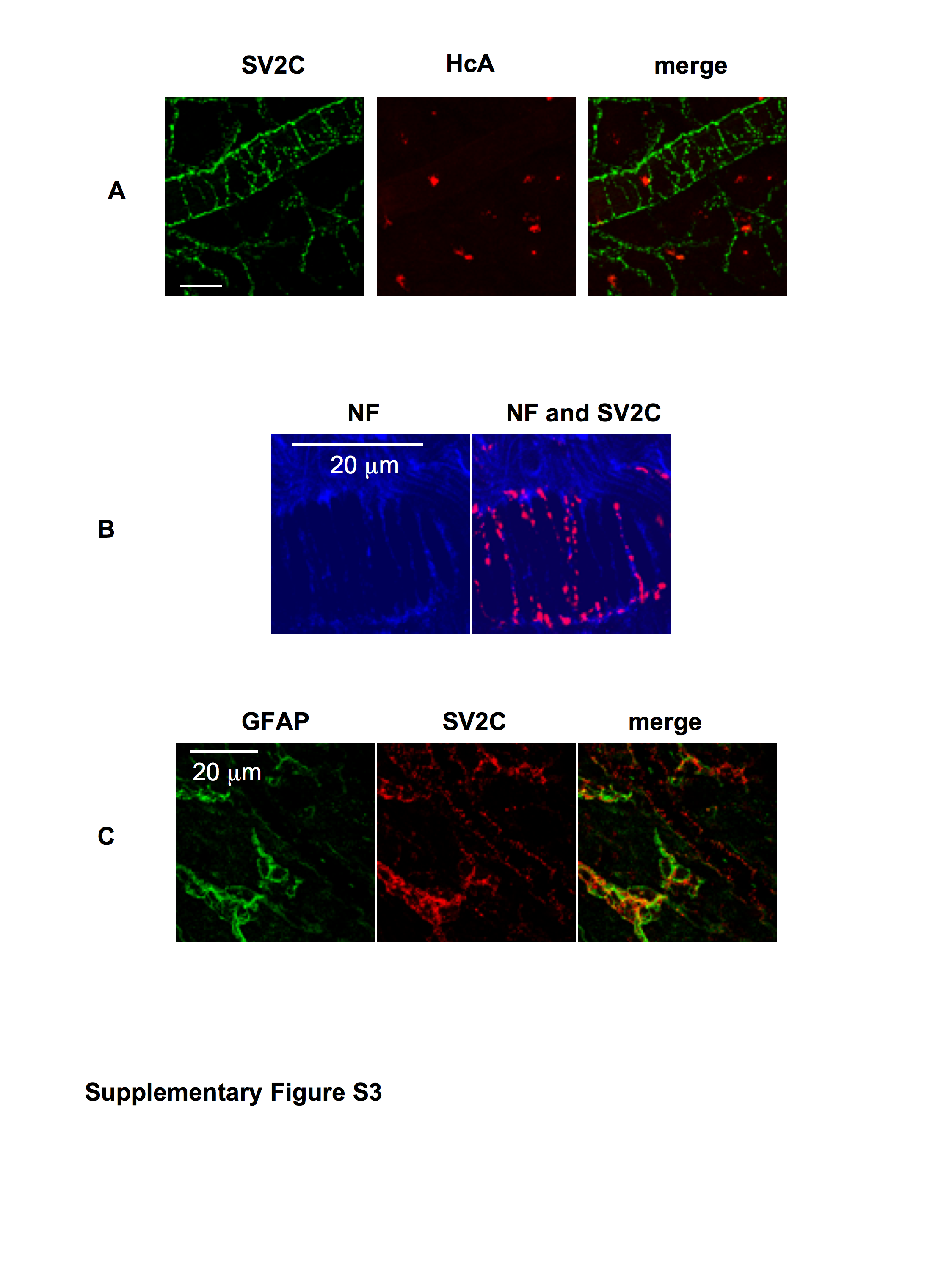

Supplement: Figure S3 — Immunolabeling pattern of SV2C in intestinal submucosa. (A) HcA (red) was injected into the lumen of a ligated intestinal loop and incubated for 1 h at room temperature. A network of thin cell extensions around microvessels in the submucosa were stained with anti-SV2C (green), and not with HcA. HcA only decorated some filament extremities. (B) Thin neuronal extensions around microvessels were labeled with anti-neurofilament (NF, blue) and anti-SV2C (red) antibodies. (C) Glial cells in the submucosa were stained with anti-GFAP (green) and anti-SV2C (red), but not with HcA (not shown) (scale bar = 20 µm). (TIF) [file ppat.1002583.s003.tif]
